# NDRG2 suppression as a molecular hallmark of photoreceptor-specific cell death in the mouse retina

**DOI:** 10.1038/s41420-018-0101-2

**Published:** 2018-09-12

**Authors:** Cheng-Biao Hu, Bing-Dong Sui, Bao-Ying Wang, Gao Li, Cheng-Hu Hu, Chen-Xi Zheng, Fang-Ying Du, Chun-Hui Zhu, Hong-Bo Li, Yan Feng, Yan Jin, Xiao-Rui Yu

**Affiliations:** 1 Department of Biochemistry and Molecular Biology, School of Basic Medical Sciences, Xi’an Jiaotong University Health Science Center, 710061 Xi’an, Shaanxi China; 2 Key Laboratory of Environment and Genes Related to Diseases (Xi’an Jiaotong University), Ministry of Education, Xi’an, Shaanxi Sheng China; 3 State Key Laboratory of Military Stomatology, Center for Tissue Engineering, Fourth Military Medical University, 710032 Xi’an, Shaanxi China; 4 Xi’an Institute of Tissue Engineering and Regenerative Medicine, 710032 Xi’an, Shaanxi China; 5 Department of Stomatology, The People’s Hospital of Zhangqiu City, 250200 Zhangqiu, Shandong China

## Abstract

Photoreceptor cell death is recognized as the key pathogenesis of retinal degeneration, but the molecular basis underlying photoreceptor-specific cell loss in retinal damaging conditions is virtually unknown. The N-myc downstream regulated gene (NDRG) family has recently been reported to regulate cell viability, in particular NDRG1 has been uncovered expression in photoreceptor cells. Accordingly, we herein examined the potential roles of NDRGs in mediating photoreceptor-specific cell loss in retinal damages. By using mouse models of retinal degeneration and the 661 W photoreceptor cell line, we showed that photoreceptor cells are indeed highly sensitive to light exposure and the related oxidative stress, and that photoreceptor cells are even selectively diminished by phototoxins of the alkylating agent *N*-Methyl-*N*-nitrosourea (MNU). Unexpectedly, we discovered that of all the NDRG family members, NDRG2, but not the originally hypothesized NDRG1 or other NDRG subtypes, was selectively expressed and specifically responded to retinal damaging conditions in photoreceptor cells. Furthermore, functional experiments proved that NDRG2 was essential for photoreceptor cell viability, which could be attributed to NDRG2 control of the photo-oxidative stress, and that it was the suppression of NDRG2 which led to photoreceptor cell loss in damaging conditions. More importantly, NDRG2 preservation contributed to photoreceptor-specific cell maintenance and retinal protection both in vitro and in vivo. Our findings revealed a previously unrecognized role of NDRG2 in mediating photoreceptor cell homeostasis and established for the first time the molecular hallmark of photoreceptor-specific cell death as NDRG2 suppression, shedding light on improved understanding and therapy of retinal degeneration.

## Introduction

Photoreceptor cell death is recognized as a major contributor to visual impairment in retinal degeneration such as age-related macular degeneration (AMD) and the hereditary retinitis pigmentosa (RP), two leading causes of blindness in the modern world with only few available treatments^1–3^. Current pathophysiological understanding of photoreceptor cell damages has highlighted etiological significance of optical and chemical stimuli, as well as genetic alterations, which initiate photoreceptor cell apoptosis through mechanisms including multiple pathways and transcriptional regulations^4,5^. Especially, it is notable that among all the retinal cell types, photoreceptor cells are particularly sensitive to light exposure and the induced oxidative stress, despite the possibly affected retinal ganglion cells (RGCs), and that photoreceptor cells are even selectively diminished by certain phototoxins, such as the DNA alkylating agent *N*-Methyl-*N*-nitrosourea (MNU)^6–8^. However, the molecular basis underlying this photoreceptor-specific cell loss is still unknown, the elucidation of which will provide a clearer and more complete comprehension of retinal damages that benefits approaches to retinal degeneration.

The N-myc downstream regulated gene (NDRG) family of proteins, which consists of 4 members denoted as NDRG1–4, are well conserved factors originally reported to participate in tumorigenesis and metastasis^9–11^. Emerging evidence has further uncovered the importance of NDRGs in regulating cellular viability and various behaviors, in particular NDRG1–3 have been revealed to confer apoptotic protection effects in response to cellular stress conditions of oxidative and alkylating damages^12–14^. Accumulating data also suggest organ/tissue-specific biological function of NDRGs in development and postnatal homeostasis, among which NDRG1, 2, and 4 are widely distributed in the nervous system and are involved in neural degenerative diseases^15–17^. Particularly, for the specialized neurons in the vertebrate retina, which include rod and cone photoreceptors, interneurons, and RGCs^18^, only NDRG1 has been documented expression with contributions to morphology in zebrafish photoreceptors, while NDRG2 was detected in murine astrocytes except the proliferative retinal Müller glia^19,20^. At the present time, it is unclear as to whether existence and altered level(s) of certain NDRG subtype(s) correlate with functional outcomes in retinal damaging conditions, and whether the effects are cell-type specific.

In this study, we aimed to establish the molecular characteristics of photoreceptor-specific cell death in retinal degeneration with regard to expression and function of the NDRG family. We intended to address the following issues: (1) Whether and which NDRG family member(s) specifically respond to photoreceptor cell damages; (2) Whether modulation of the certain NDRG(s) preserves photoreceptor cell viability in damaging conditions; and (3) Whether alterations of the NDRG candidate(s) contribute to the development and treatment of retinal degeneration induced by photoreceptor-specific cell death. On the basis of light exposure/oxidative stress-provoked and MNU-provoked retinal damaging models both in vitro and in vivo, we hereby provided that decline of NDRG2, but not the originally hypothesized NDRG1 or other NDRG subtypes, characterized photoreceptor-specific cell loss in the mouse retina. Our results unraveled for the first time the specific molecular hallmark of photoreceptor cell death, shedding light on improved understanding and therapy of retinal degeneration.

## Results

### Photoreceptor cells are highly susceptible to damages in retinal degeneration

**Fig. 1:**
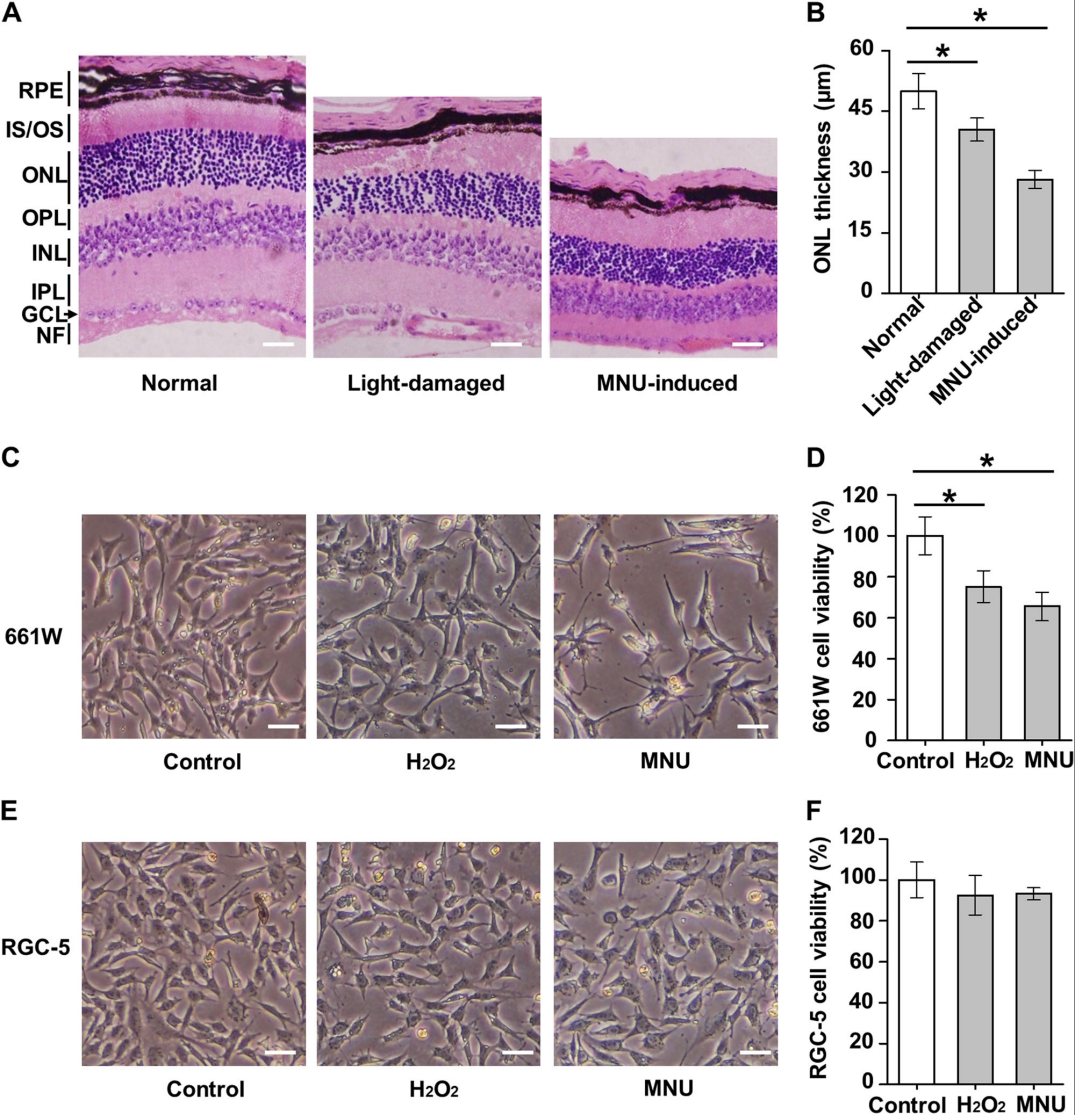
Responses to retinal damaging factors in retinal tissues and cells. **a**, **b** Representative H&E staining of retinal tissues (**a**) and statistical analysis of thickness of the outer nuclear layer (ONL, representing photoreceptor cell bodies) (**b**). Scale bar = 20 μm. *N* = 4~5 per group. RPE retinal pigment epithelium, IS/OS inner segment/outer segment, OPL outer plexiform layer, INL inner nuclear layer, IPL inner plexiform layer, GCL ganglion cell layer, NF nerve fiber, MNU *N*-Methyl-*N*-nitrosourea. **c**, **d** Cell morphology (**c**) and viability analysis (**d**) by methyl thiazolyl tetrazolium (MTT) of the 661 W photoreceptor cell line. **e**, **f** Cell morphology (**e**) and viability analysis (**f**) by MTT of the retinal ganglion cell-5 (RGC-5) cell line. Scale bar = 5 μm. *N* = 3 per group. H_2_O_2_, hydrogen peroxide. Data represents mean ± SD. *P < 0.05

To investigate the molecular hallmark(s) of photoreceptor-specific cell death, we firstly established retinal degeneration models in mice characterized by photoreceptor cell loss. Extensive studies have indicated that light exposure is involved in the progress of retinal degeneration and that light-induced retinal damages in animals are generally similar to those seen in human AMD patients^21^. In this study, we confirmed previous findings that correspondent with its physiological function of sensing photons^22^, photoreceptor cells are sensitive to the light exposure pressure, as demonstrated by decline of the thickness of the outer nuclear layer (ONL) in the light-damaged mouse retina, where the bodies of photoreceptor cells reside^18^ (Fig. 1a, b). To more specifically induce photoreceptor cell loss, we have also applied the selective phototoxin MNU^8^ and detected sharp ablation of the retinal ONL thickness in the treated mice (Fig. 1a, b).

To build up photoreceptor-specific cell death conditions in vitro mimicking the in vivo changes, we used a mouse retinal cell line with photoreceptor origin, 661 W^23^, and examined its response to the retinal damaging factors. The photo-oxidative stress has received significant attention as the key pathogenic contributor to retinal damages induced by light exposure, which is predisposed to produce reactive oxygen species (ROS) in the retinal tissue microenvironment with high oxygen tension^24^. We provided here that both the hydrogen peroxide (H_2_O_2_) and MNU triggered loss of 661 W cell viability, particularly MNU with remarkable impacts, as indicated by the morphological observation (Fig. 1c, d). Furthermore, the loss of 661 W cell viability could be attributed to provoked cell death by H_2_O_2_ and MNU, as shown by increased positively stained proportion of propidium iodide (PI) for dead cells^25,26^ (Supplementary Fig. 1A). However, despite recent controversies regarding the RGC origin of this cell line^27^, RGC-5 cells exhibited lower degree of susceptivity to both H_2_O_2_-induced and MNU-induced damages, consistently with previous results that these cells have damage-resistant features not part of the characteristics of the 661 W cells^28^ (Fig. 1e, f; Supplementary Fig. 1B). The above findings collectively suggested that photoreceptor cells are indeed highly susceptible to damages in retinal degeneration.

### NDRG1 is generally suppressed in retinal cells under damaging conditions

**Fig. 2:**
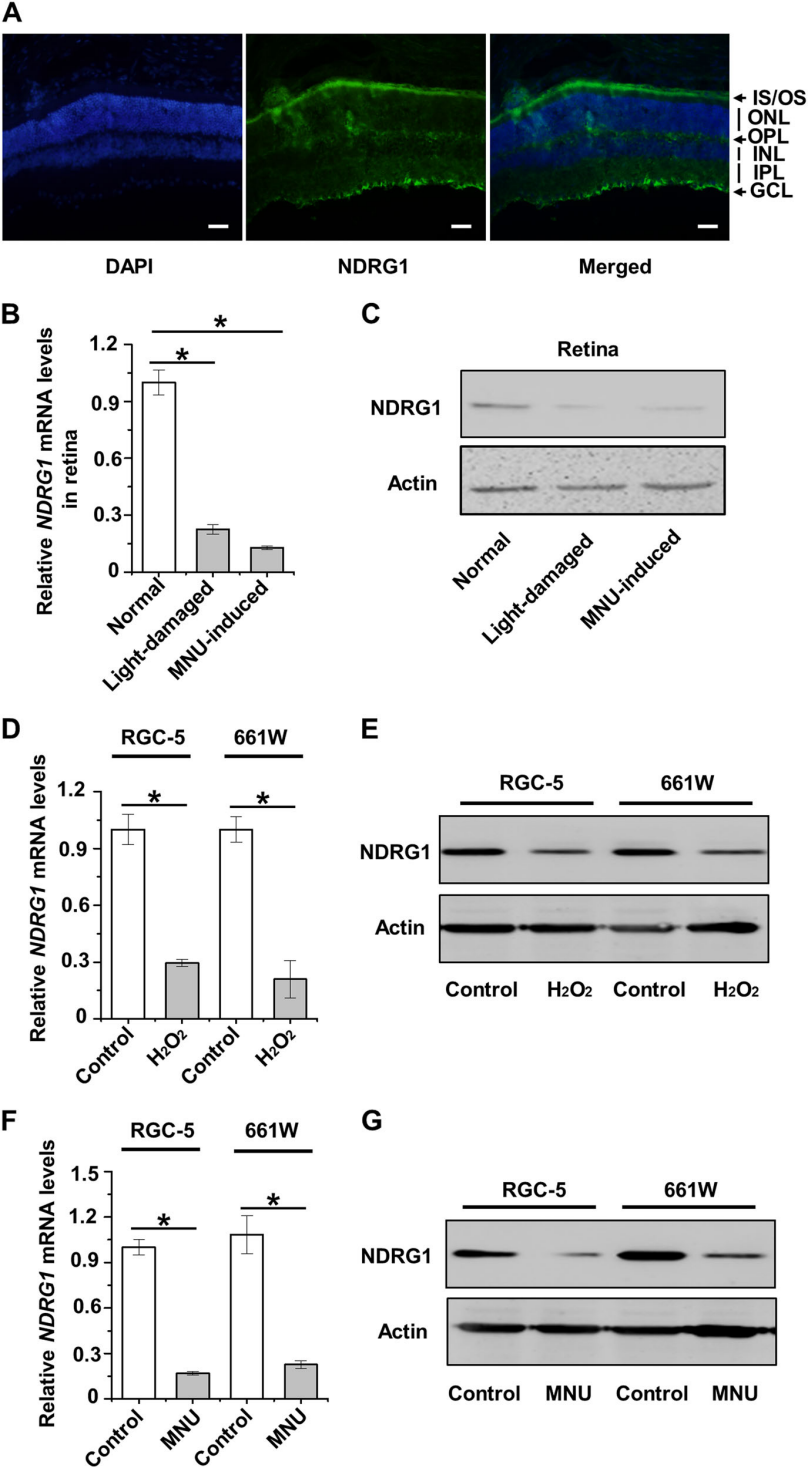
Involvement of N-myc downstream regulated gene 1 (NDRG1) in retinal damages. **a** Immunofluorescent staining of NDRG1 in the retinal tissue. Scale bar = 20 μm. **b**, **c** Quantitative real time polymerase chain reaction (qRT-PCR) analysis of the mRNA expression level (**b**) and western blot analysis of the protein expression level (**c**) of NDRG1 in the retinal tissue. **d–g** mRNA and protein expression levels of NDRG1 in the 661 W and RGC-5 cell lines. *N* = 3 per group. Data represents mean ± SD. *P < 0.05

To explore whether the NDRG family member(s) contribute to photoreceptor-specific cell loss, we originally hypothesized that NDRG1 was the putative candidate, considering that NDRG1 is the only NDRG subtype currently found expression in the retina, and that NDRG1 is involved in forming a normal morphological shape in zebrafish photoreceptors^19^. Accordingly, we indeed detected major distribution of NDRG1 in the inner segment/outer segment (IS/OS) of photoreceptor cells^29^, while NDRG1 was also present in the inner plexiform layer (IPL)/ganglion cell layer (GCL), as well as the outer plexiform layer (OPL) (Fig. 2a), respectively, indicating its expression in RGCs and interneurons/Müller glia^18^. Furthermore, mRNA expression levels of NDRG1 in the mouse retina were significantly reduced by light and MNU treatments, associated with declined protein expression levels (Fig. 2b, c). These results supported participation of NDRG1 in retinal degeneration.

Nevertheless, by comparing NDRG1 alterations in RGC-5 and 661 W cell lines, we found that NDRG1 did not specifically characterize photoreceptor cells in damage conditions. As demonstrated, H_2_O_2_ treatment inhibited mRNA and protein expression levels of RGC-5 and 661 W cells to similar extents (Fig. 2d, e). Furthermore, although that MNU selectively induced 661 W cell death (Fig. 1c, d), NDRG1 expression in both RGC-5 and 661 W cells was suppressed (Fig. 2f, g). These data suggested that decline of NDRG1 was not a specific marker of photoreceptor cell death.

### NDRG2 represents the specifically expressed NDRG family member in photoreceptor cells

**Fig. 3:**
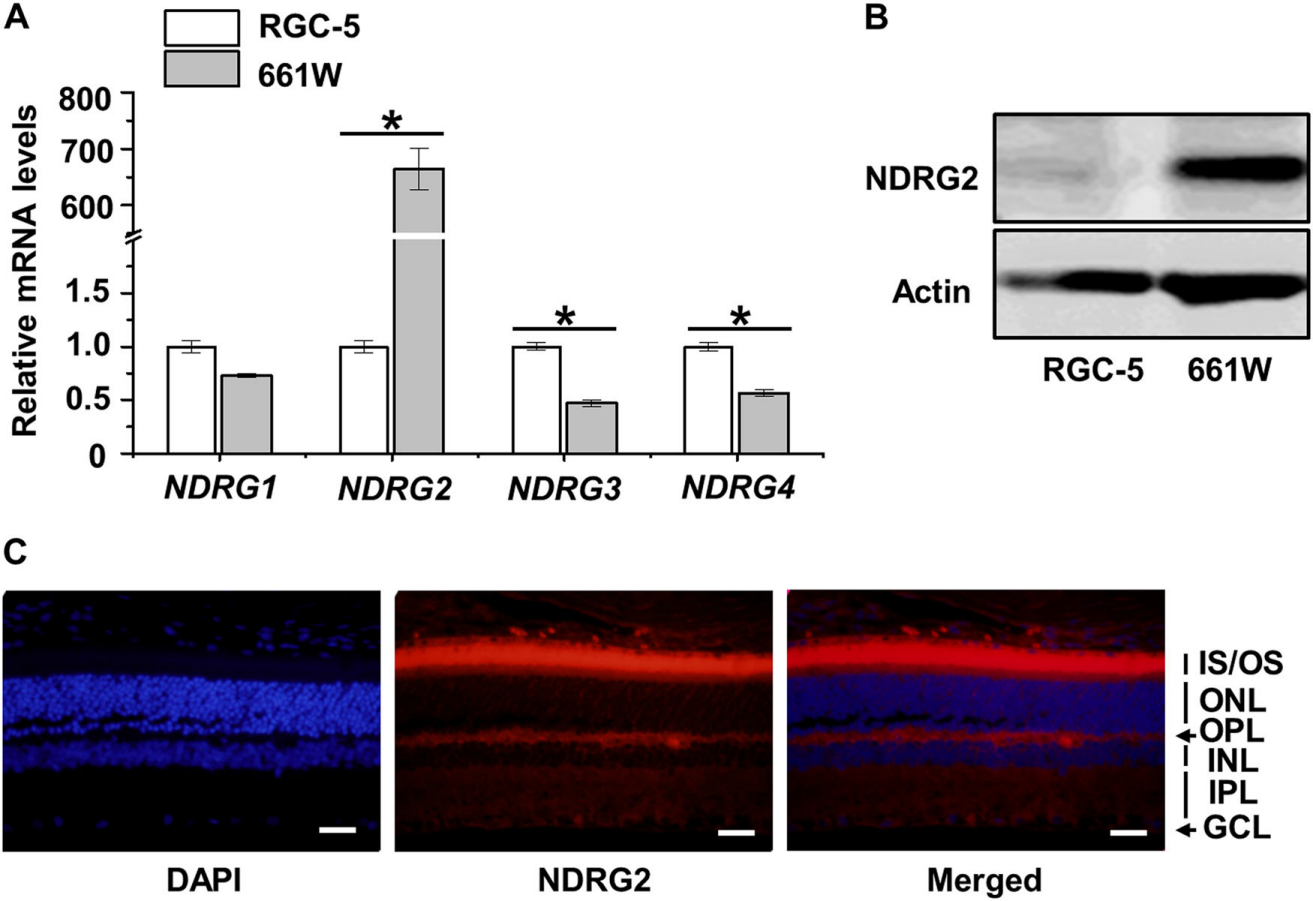
Expression of NDRG family members in retinal tissues and cells. **a** qRT-PCR analysis of the mRNA expression levels of NDRG family members in the 661 W and RGC-5 cell lines. **b** Western blot analysis of the protein expression level of NDRG2 in the 661 W and RGC-5 cell lines. **c** Immunofluorescent staining of NDRG2 in the retinal tissue. Scale bar = 20 μm. *N* = 3 per group. Data represents mean ± SD. *P < 0.05

Given the above analysis on NDRG1, we continued to examine whether other NDRG family members are expressed specifically in the retina with potential function to regulate cell death. Quantitative real-time polymerase chain reaction (qRT-PCR) screening on the 4 subtypes of NDRGs demonstrated that only NDRG2 was expressed substantially higher in 661 W cells compared to RGC-5 cells, with over 600-fold mRNA level changes (Fig. 3a). The specific expression of NDRG2 in 661 W cells was further confirmed in the protein level, for NDRG2 protein was barely detected in RGC-5 cells (Fig. 3b). Moreover, in vivo data of the mouse retina showed primary location of NDRG2 expression in the photoreceptor IS/OS, despite slight expression in OPL and IPL (Fig. 3c). These findings indicated that NDRG2 represents the specifically expressed NDRG family member in photoreceptor cells.

### NDRG2 specifically declines in photoreceptor cells in responses to damages

**Fig. 4:**
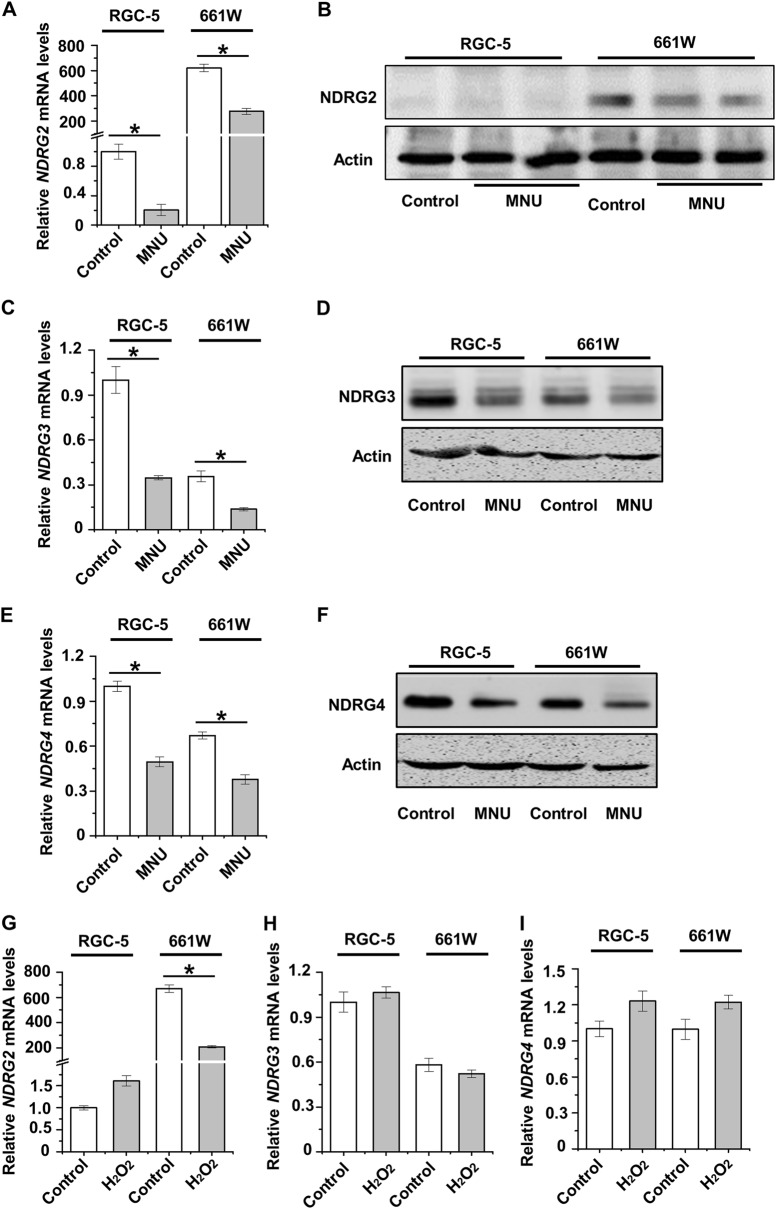
Involvement of NDRG2–4 in retinal cell damages. **a**, **b** qRT-PCR analysis of the mRNA expression level (**a**) and western blot analysis of the protein expression level (**b**) of NDRG2 in the 661 W and RGC-5 cell lines. **c**, **d** qRT-PCR analysis of the mRNA expression level (**c**) and western blot analysis of the protein expression level (**d**) of NDRG3 in the 661 W and RGC-5 cell lines. **e**, **f** qRT-PCR analysis of the mRNA expression level (**e**) and western blot analysis of the protein expression level (**f**) of NDRG4 in the 661 W and RGC-5 cell lines. **g–i** qRT-PCR analysis of the mRNA expression levels of NDRG2–4 in the 661 W and RGC-5 cell lines. *N* = 3 per group. Data represents mean ± SD. *P < 0.05

We next examined whether NDRG2, or perhaps NDRG3 and NDRG4 as well, specifically participated in photoreceptor cell damages. After the selective phototoxin MNU treatment, compared to the basal mRNA expression level in RGC-5 cells, NDRG2 mRNA expression in 661 W cells underwent a sharp decrease from approximately 600 fold to about 200 fold (Fig. 4a). Suppression of NDRG2 by MNU in 661 W cells was confirmed in the protein level, while NDRG2 protein expression remained scarce in RGC-5 cells (Fig. 4b). Expression of NDRG3 and NDRG4, however, was generally inhibited in RGC-5 and 661 W cells under MNU stress (Fig. 4c-f). Furthermore, for H_2_O_2_-driven oxidative damages, only NDRG2 was specifically suppressed in 661 W cells (Fig. 4g-i). The down-regulation of NDRG2 in the cytoplasm of 661 W cells in retinal damaging conditions was also revealed by immunocytochemistry (Supplementary Fig. 2). These data indicated that NDRG2 specifically declines in photoreceptor cells in responses to damaging stresses.

### NDRG2 maintains photoreceptor cell viability and counteracts oxidative stress

**Fig. 5:**
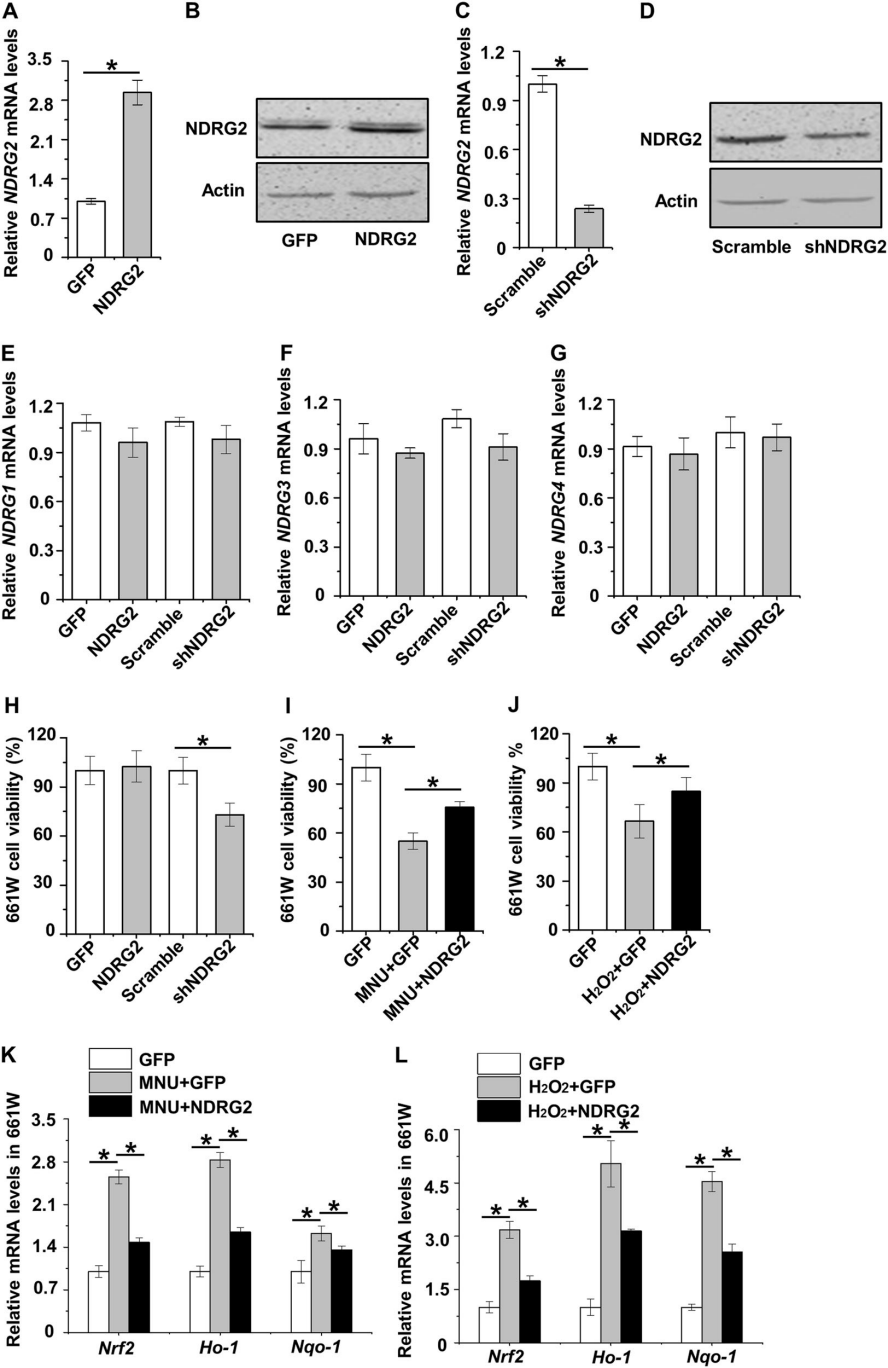
Function of NDRG2 in maintaining photoreceptor cell viability and counteracting oxidative stress. **a**, **b** qRT-PCR analysis of the mRNA expression level (**a**) and western blot analysis of the protein expression level (**b**) of NDRG2 in the 661 W cell line. 661 W cells were transduced with either green fluorescent protein (GFP)-based lentiviral vector for NDRG2 overexpression (the NDRG2 group) or the vacant vector (the GFP group). **c**, **d** qRT-PCR analysis of the mRNA expression level (**c**) and western blot analysis of the protein expression level (**d**) of NDRG2 in the 661 W cell line. 661 W cells were transfected with either a short heparin RNA (shRNA) for NDRG2 knockdown (the shNDRG2 group) or the scrambled shRNA (the scramble group). **e-g** qRT-PCR analysis of the mRNA expression levels of NDRG1, 3 and 4 in the 661 W cell line upon NDRG2 manipulations. **h-j** MTT analysis of cell viability of the 661 W cell line upon NDRG2 manipulations. **k**, **l** qRT-PCR analysis of the mRNA expression levels of the antioxidant master transcription factor Nuclear factor E2-related factor 2 (Nrf2), the regulated antioxidant genes Heme oxygenase-1 (Ho-1) and NAD(P)H:quinone oxidoreductase-1 (Nqo-1) in the 661 W cell line upon NDRG2 manipulations. *N* = 3 per group. Data represents mean ± SD. *P < 0.05

Next, we evaluated function of NDRG2 in regulating photoreceptor cell viability. By using a green fluorescent protein (GFP)-containing lentivirus vector, we established the NDRG2-overexpression tool and successfully up-regulated both mRNA and protein levels of NDRG2 after transfection into 661 W cells (Fig. 5a, b). Furthermore, we applied a short hairpin RNA (shRNA) for NDRG2 and partially silenced NDRG2 expression in 661 W cells (Fig. 5c, d). Importantly, neither NDRG2 overexpression nor silencing influenced the levels of NDRG1, 3, and 4 in 661 W cells, which proved the specificity of NDRG2 intervention (Fig. 5e-g). With regard to the photoreceptor cell viability, data demonstrated that NDRG2 overexpression in the basal condition did not regulate 661 W cell viability, while down-regulation of NDRG2 exerted detrimental effects (Fig. 5h). Significantly, NDRG2 overexpression under MNU treatment partially maintained 661 W cell viability, indicating NDRG2 opposes photoreceptor damages (Fig. 5i). The NDRG2 protection on photoreceptor viability was further confirmed under H_2_O_2_-induced oxidative damages, where NDRG2 overexpression prevented 661 W cell loss to some extent (Fig. 5j). These findings suggested that down-regulation of NDRG2 in responses to retinal damaging factors mediates photoreceptor cell loss and damages.

Then, we intended to provide clues of how NDRG2 protected photoreceptor cells under MNU and H_2_O_2_ damages. Notably, despite its well-known role as an alkylating agent^8^, MNU induction on DNA damages and cell apoptosis could also be attributed to its trigger of oxidative stress^30^. Accordingly, we indeed discovered that amelioration of ROS contents in photoreceptor cells served as a common reason underlying NDRG2 protection on 661 W cell viability under both MNU and H_2_O_2_ treatments (Supplementary Fig. 3). Furthermore, NDRG2 alleviation of photoreceptor oxidative stress resulted in restriction of the stimulated photoreceptor antioxidant defense responses under damaging conditions, as shown by inhibited mRNA expression levels of the antioxidant master transcription factor Nuclear factor E2-related factor 2 (Nrf2)^31^ and the regulated antioxidant genes Heme oxygenase-1 (Ho-1)^32^ and NAD(P)H:quinone oxidoreductase-1 (Nqo-1)^33^ (Fig. 5k, l). These data indicated that counteraction of oxidative stress plays a part in the underlying mechanism of NDRG2 protection on photoreceptor cell viability.

### NDRG2 contributes to photoreceptor cell loss and protection in the mouse retina

**Fig. 6:**
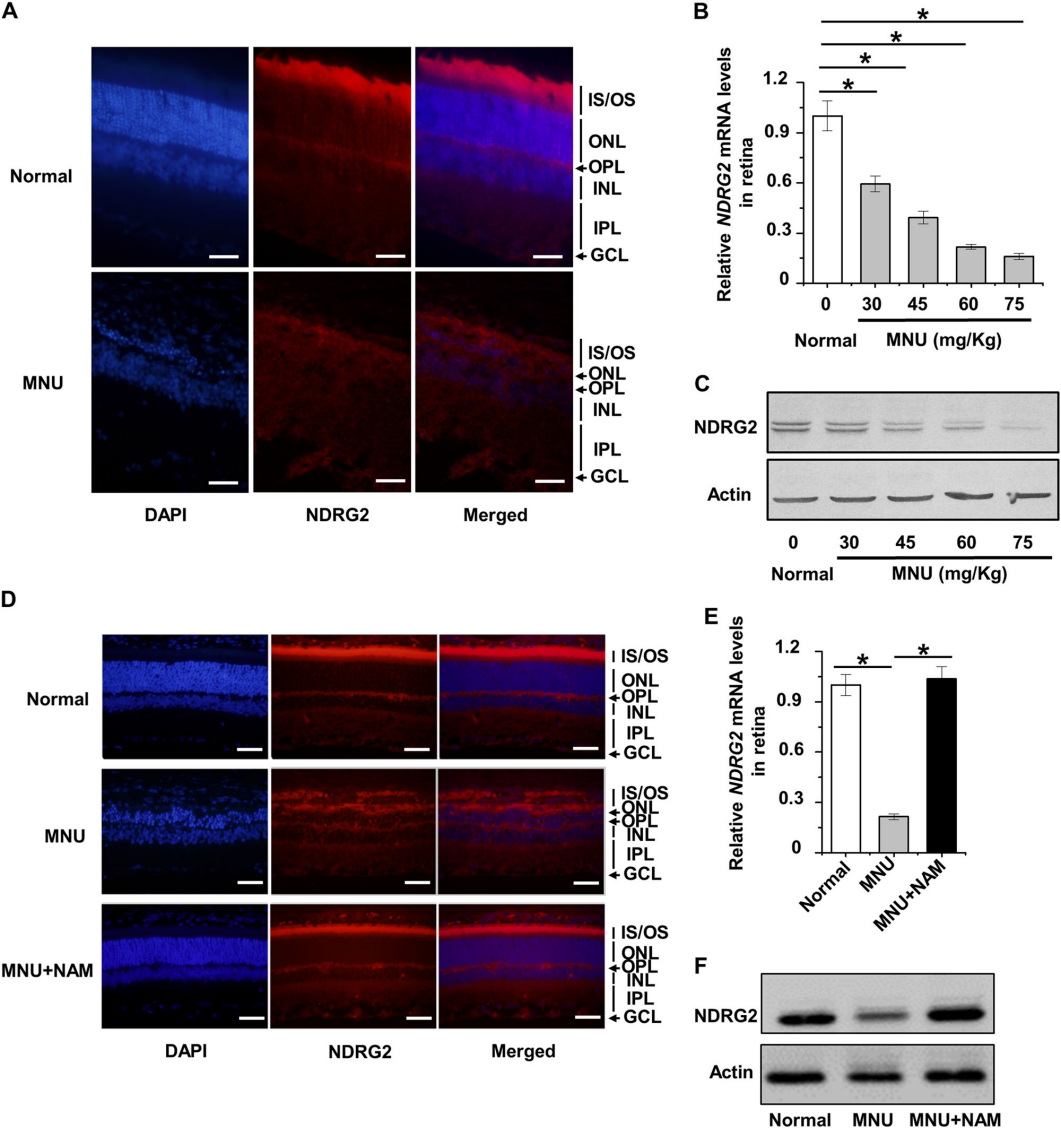
Participation of NDRG2 in retinal photoreceptor cell loss and protection. **a** Immunofluorescent staining of NDRG2 in the retinal tissue. Scale bar = 20 μm. **b**, **c** qRT-PCR analysis of the mRNA expression level (**b**) and western blot analysis of the protein expression level (**c**) of NDRG2 in the retinal tissue. *N* = 6~7 per group. **d** Immunofluorescent staining of NDRG2 in the retinal tissue. Scale bar = 20 μm. NAM, nicotinamide. **e**, **f** qRT-PCR analysis of the mRNA expression level (**e**) and western blot analysis of the protein expression level (**f**) of NDRG2 in the retinal tissue. *N* = 5 per group. Data represents mean ± SD. *P < 0.05

The above results prompted us to examine the role of NDRG2 in photoreceptor-specific cell death in vivo. On the basis of MNU-induced retinal degeneration model caused by photoreceptor-specific cell loss, as confirmed again by the dramatic disruption of ONL morphology, we discovered that expression of NDRG2 in the photoreceptor IS/OS layers was specifically reduced, to an extent that is almost comparable to the background level (Fig. 6a). The suppression of NDRG2 in photoreceptor cells led to declined NDRG2 expression in the whole retina tissue, and the decrease was dose-dependent on MNU in both mRNA and protein levels (Fig. 6b, c). Furthermore, considering that NDRG2 could be translocated into nuclei upon cell stress^34^, we also examined the nuclear protein expression level and found that a small amount of NDRG2 protein was indeed imported (Supplementary Fig. 4). These results collectively indicated that NDRG2 suppression in photoreceptor cells contributed to degeneration of the mouse retina.

Finally, we aimed to evaluate whether NDRG2 preservation contributes to protection of retina in damaging conditions. Nicotinamide (NAM), a water-soluble B-group vitamin (vitamin B_3_), has been well documented to block MNU-induced photoreceptor cell loss and retinopathy in rodents^35–37^. Accordingly, we confirmed in the present study that NAM treatment completely blocked detrimental effects of MNU on the mouse retina, with preserved expression of NDRG2 in the photoreceptor IS/OS layers (Fig. 6d). As further demonstrated in the whole retinal mRNA and protein levels, NDRG2 expression was totally rescued by NAM, underlying the maintained photoreceptor cell viability and the protection of the mouse retina (Fig. 6e, f). These findings suggested that NDRG2 preservation was indeed of importance in retinal homeostasis.

## Discussion

Photoreceptor cell death is well documented as the key pathogenesis of retinal degeneration, but the molecular basis underlying the distinguished sensitivity of photoreceptor cells to damaging factors including light and chemical stimuli is virtually unknown^1,4,5^. In this study, we examined the potential roles of NDRGs, a family with wide distribution in the nervous system^15–17^ and putative function to regulate cell viability^12–14^, in mediating photoreceptor-specific cell loss. We discovered that of all the NDRG family members, NDRG2, but not the originally hypothesized NDRG1 or other NDRG subtypes, was selectively expressed and specifically responded to retinal damaging conditions in photoreceptor cells. Furthermore, functional experiments proved that it was the suppression of NDRG2 by both H_2_O_2_ and MNU that led to photoreceptor cell loss. More importantly, NDRG2 preservation contributed to photoreceptor cell maintenance and retinal protection in damaging conditions, which could be attributed to NDRG2 amelioration on the photo-oxidative stress. Our findings for the first time established the molecular hallmark of photoreceptor-specific cell death, shedding light on improved understanding and therapy of retinal degeneration.

The mammalian retina is a complex array of specialized neurons and glia that converts and encodes light energy into electrical impulses to begin the visual process^18^. Pathophysiologically, despite multiple genetic mutations, it is the damages of photoreceptor cells by prolonged light activation or chemical insults that contribute to most cases of blindness in retinal degeneration^1^. Current mechanistic studies of photoreceptor cell death have revealed the importance of several key processes, including the increased permeability of cation channels^38^, disordered intracellular calcium homeostasis^39^, the occurrence of oxidative stress^30^, changes in autophagic events^40^, and finally the triggering of apoptosis via pathways dependent or independent on caspase^22^ and transducin^41^, the dying photoreceptor cells during which released chemokines to activate microglia^42^ and purines to induce death of neighboring photoreceptors^43^ thus further aggravating the cell loss. However, although remarkable advance has been achieved in understanding the mechanisms of photoreceptor cell death per se, almost all of the above processes also contribute to impaired viability of other cells such as RGCs in various damaging conditions^44–46^. At the present time, compared to RGCs, the particular sensitivity of photoreceptor cells to light exposure and selective phototoxins including MNU has been noticed^6–8^, but the putative molecular basis is still lacking. In this study, we provided for the first time the hallmark of photoreceptor-specific cell death, NDRG2, which selectively expressed and specifically responded to retinal damaging factors in photoreceptor cells. Our findings help to establish a clearer, more complete deciphering of the distinguished characteristics of photoreceptor cell loss in retinal damages.

The NDRG family of proteins, originally known as tumor suppressors^9–11^, have been uncovered expression across developmental and the postnatal organism in an organ/tissue specific pattern, especially in the nervous system^15–17^. Particularly, NDRG1 is expressed in the photoreceptor layer of the developing retina and contributes to photoreceptor OS formation in zebrafish^19^, while NDRG2 was previously reported absence in the retinal Müller glia^20^. Here, we screened and surprisingly found that it is NDRG2 that represents the major NDRGs in photoreceptors and is specifically expressed in the mouse photoreceptor cells. This differential expression patterns of NDRG family proteins in the retina are also prominent in the whole nervous system, where each NDRG may play a partially redundant role in specific cells in the brain^47^. Furthermore, for a certain NDRG family member such as NDRG1, differential expression has also been revealed between even rod and cone photoreceptor cells in carp^48^. For the upstream regulation, *NDRG* genes are transcriptionally repressed by Myc, a master switch for cell proliferation and differentiation, but the regulation by Myc constitutes one common mechanism among different NDRG family members^49,50^. However, each *NDRG* gene is transcribed into multiple isoforms with distinct mRNAs and proteins, despite the shared 57–65% amino acid identity across family members^9^. Therefore, although each *NDRG* gene might still be transcriptionally regulated by distinguished factors^12,51^, epigenetic^52^, and posttranscriptional regulations^53,54^ are assumed to participate in the expression level determination of NDRGs in responses to certain environmental stimuli. The very reason underlying specific expression of NDRG2 in photoreceptor cells remains to be elucidated in future studies.

One particular clue that may contribute to specific regulation of NDRG2 in photoreceptor cells lies in the functional need of NDRG2 to modulate photoreceptor cell viability in variable visual conditions. Indeed, we detected high sensitivity of NDRG2 to both optical/oxidative and chemical stimuli, upon which suppression of NDRG2 mediated loss of photoreceptor cells. Previously, NDRG2 has also been documented protection against H_2_O_2_-induced apoptosis of skeletal muscle cells, in which NDRG2 ameliorated endoplasmic reticulum (ER) stress, reduce cleavage of caspase-3, and poly (ADP-ribose) polymerase (PARP), inhibited expression of pro-apoptotic Bax while enhanced the pro-survival Bcl-2 and Bcl-xL protein levels^13^. Beyond these mechanisms collectively to prevent apoptosis also in photoreceptor cells^41^, in the present study, we further proved that NDRG2 functions fundamentally to alleviate oxidative stress in photoreceptor cells under both H_2_O_2_-provoked and MNU-provoked damages. The mechanism of NDRG2 to alleviate oxidative stress should be attributed to direct scavenging of cellular ROS contents, but not stimulation of the antioxidant defense system, as shown by our data that expression of the antioxidant genes merely correlated with ROS density in contrast to NDRG2 levels in photoreceptor cells. Besides, NDRG2 protection on photoreceptor cell viability might also be due to mechanisms such as maintained autophagy, for which certain NDRG is involved in the autophagic mammalian target of rapamycin (mTOR) signaling-determined tumor resistance toward alkylating chemotherapy^12^. We have additionally found that deprivation of serum in culture of 661 W cells, which stimulated autophagic reactions^55^, offered protection against MNU-induced damages (unpublished data). The molecular pathways underlying NDRG2 scavenging of ROS and potential contributions of other protective mechanisms in photoreceptor cells should be explored in the future.

The most important finding of the current study is to unravel NDRG2 as the molecular hallmark of photoreceptor-specific cell viability, which was confirmed not only in vitro but also in vivo in retinal degeneration and treatment. In fact, there is a multitude of treatment strategies and compounds that at least partially prevent retinal degeneration in animal models, including the calcium channel blocker D-diltiazem^56,57^, various antioxidants^24,58^, caspase inhibitors^59,60^, multiple neuroprotective agents including NAM^35,36^ and other neurotrophic cytokines^61^, apoptotic gene therapies^62,63^, and the recent stem cell transplantation^64,65^. Nevertheless, while retinal degeneration in preclinical studies could be effectively prevented, there does not seem to be a single treatment available at present that rescues photoreceptor cell damages in human^66,67^. Here, by using MNU-induced mouse models of retinal degeneration and NAM-based treatment, we proposed that specifically preservation of NDRG2 in photoreceptor cells contributes to maintenance of retinal homeostasis, paving an avenue for feasible targeted therapies in context of reducing the sensitivity of photoreceptor cells to retinal damaging factors in vivo. Actually, previous proof-of-concept reports have established interfering approaches to slow down the visual cycle based on rhodopsin inhibition^68,69^, but the selective molecular intervention strategies on photoreceptor cells were not provided. Based on our findings, despite pharmacological agents of NDRG2 modulators await to be clarified, genetic overexpression of photoreceptor NDRG2 based on cell-targeting techniques such as the aptamer-modified liposomes^70,71^ may represent a promising solution to prevent and rescue retinal degeneration, which is worth to be evaluated by further experiments.

In summary, NDRG2 contributes to photoreceptor cell homeostasis, and NDRG2 suppression serves as a molecular hallmark of photoreceptor-specific cell death in the mouse retina. These findings shed light on improved understanding and therapy of retinal degeneration.

## Materials and Methods

### Animals

All experiments were approved by Xi’an Jiaotong University and were performed following the Guidelines of Intramural Animal Use and Care Committee of Xi’an Jiaotong University. Animal experiments were also performed following the ARRIVE guidelines.

The 12-week-old male C57BL/6 mice (weight, 22–25 g) (Laboratory Animal Center, Xi’an Jiaotong University, China) were used and were randomly assigned to experimental groups. The light exposure-induced retinal damaging model was established based on the published protocol with modifications^22^. Briefly, mice were exposed to 5000-lux white light for 24 h followed by a 3-day dark recovery before sacrifice. For the MNU-provoked retinal damaging model, accordingly^37^, MNU (Sigma-Aldrich, USA) was injected intraperitoneally once at typically 60 mg/kg and were sacrificed after 3 days. The dosage of MNU varies at 30, 45, 60, and 75 mg/kg at certain experiments. For NAM-mediated rescue of MNU-induced retinopathy, accordingly^37^, NAM (Sigma-Aldrich, USA) was subcutaneously administrated at 50 mg/kg immediately after MNU injection. The mice were maintained with good ventilation and a 12-h light/dark cycle, and were kept feeding and drinking ad libitum before being euthanized.

### Cell culture

The 661 W cell line was derived from mouse retinal tumors and has been characterized previously to be of cone photoreceptor cell lineage^23^. Both 661 W and RGC-5 cell lines were cultured in Dulbecco’s Modified Eagle Medium (DMEM) supplemented with 10% fetal bovine serum (FBS), 2-mM L-glutamine, 100-U/mL penicillin, and 100-g/mL streptomycin (all from Invitrogen, USA) at 37 ℃ in a humidified atmosphere of 5% CO_2_^28^. H_2_O_2_ (Sigma-Aldrich, USA) treatment was performed at 200 μM for 6 h, while MNU was applied at 400 μg/ml for 6 h.

### Lentiviral vector construction and transduction

Loss-of-function and gain-of function experiments for *NDRG2* were performed based on lentiviral vectors according to our previous report^72^. The coding region of *NDRG2* was amplified by PCR from genomic DNA using primer sequences as below: Forward, 5′-CGGACCTAAGTCAAAGGCAAG-3′; Reverse, 5′-CCCAGTGCCCTGATAACACC-3′. Target sequences for *NDRG2* shRNA were: Forward, 5′-CCGGGATGTAGGCCTCAACTATAAGCTCGAGCTTATAGTTGAGGCCTACATCTTTTTG-3′; Revers, 5′-AATTCAAAAAGATGTAGGCCTCAACTATAAGCTCGAGCTTATAGTTGAGGCCTACATC-3′. The PCR product was digested with restriction enzymes *Age*I and *Eco*RI, and inserted into the PLKO.1 vector (Thermo Fisher Scientific, USA). A lentiviral vector constructed with scrambled *NDRG2* sequence was used as the negative control. Target sequences for *NDRG2* overexpression were provided as below: Forward, 5′-GGGGTACCACCATGGCAGAACTTCAGGAG-3′; Reverse, 5′-CCGCTCGAGGAGGGTCATTCAACAGGAGAC-3′. The PCR product was digested with restriction enzymes *Eco*RI and *Bam*HI, and inserted into the pLenti6.3/V5-GW/EmGFP vector with green fluorescence (Thermo Fisher Scientific, USA). Vacant lentiviral vector was used as the negative control.

The inserted fragments were verified by Sanger sequencing (Sangon Biotech, China). Transfer vector and two packaging vectors of psPAX2 and pMD2.G (Thermo Fisher Scientific, USA) were co-transfected into 293 T cells to produce lentivirus. The lentivirus was then purified, filtered, quantified, and transduced into 661 W cells with a Calcium Phosphate Transfection System (Promega, USA). Following tests for transducing efficiency and function were performed after 24 h.

### Cell viability and death assay

Methyl thiazolyl tetrazolium (MTT)-based assay was used to determine cell viability, as stated before^73,74^. Briefly, after treatment by H_2_O_2_ or MNU, or transfected with lentiviral vectors, cells were incubated with 20-μl 5 mg/ml MTT (MP Biomedicals, USA) for 4 h. The precipitates were extracted with 180-μl DMSO and the absorbance was measured at the optical density (OD) of 490 nm as the viability index. Cell death was determined by PI staining, as previously reported^25,26^, according to manufacturer’s instruction (Sigma-Aldrich, USA).

### ROS detection

Total intracellular ROS contents were measured using the fluorescent probe Dichlorodihydrofluorescein Diacetate (DCFH)^75^. Briefly, 25-mM DCFH was added to cells at indicated time points, and was incubated for an additional 30 min at 37 ℃. After washing with PBS twice to eliminate the unlabeled DCFH, cells were examined under a fluorescence microscope (Olympus, Japan) with excitation at 488 nm.

### Immunofluorescent staining

Immunofluorescent staining of cell and tissue samples was performed according to our previous work^76,77^. For tissue samples, specimens were fixed overnight with 4% paraformaldehyde, cryoprotected with 30% sucrose, embedded in the optimal cutting temperature compound, snap-frozen, and sectioned into 15 μm sagittal sections (CM1950; Leica, Germany). For cell samples, specimens were fixed with 4% paraformaldehyde at room temperature for 30 min followed by permeabilization with 0.3% Triton-X 100 for 15 min at room temperature. Tissue and cell specimens were then blocked with 5% bovine serum antigen (BSA) (Sigma-Aldrich, USA) dissolved in PBS for 1 h at room temperature, stained overnight at 4 ℃ with primary antibodies as below: a rabbit anti-mouse/rat NDRG1 antibody (Cell Signaling Technology, USA) or a rabbit anti-mouse/rat NDRG2 antibody (Cell Signaling Technology, USA) at both concentrations of 1:100. The specimens were then stained by goat anti-rabbit-PE/FITC secondary antibodies for 1 h at room temperature at concentrations of 1:200, and were counterstained with DAPI (Sigma-Aldrich, USA).

### Retinal tissue histology

At sacrifice, retinal tissues from different experimental groups were rapidly isolated, fixed overnight with 4% paraformaldehyde, and embedded in paraffin. Five micrometer serial sections were prepared (RM2125; Leica, Germany) and underwent H&E staining for tissue histology and morphology, according to previous reports^76,78^. Quantification of ONL thickness was determined using the ImageJ 1.47 software.

### qRT-PCR analysis

Total RNA was collected from cells or retinal tissues by direct adding Trizol Reagent (Takara, Japan) and was purified by phenol-chloroform extraction. cDNA synthesis and PCR procedures were performed as described^25,26^. The primer sequences of the genes detected in this study were listed in the Supplementary Table 1. Relative expression level of each gene was obtained by normalizing against *β-actin* abundance.

### Western blot analysis

Western blot was performed as previously described^79,80^. Lysates of cells or retinal tissues were prepared using the Cell Lysis Buffer (Beyotime, China) and whole-cell proteins or nucleoproteins were, respectively, extracted. Protein samples were then loaded on sodium dodecyl sulfate-polyacrylamide gels, transferred to polyvinylidene fluoride membranes (Millipore, USA), and blocked with 5% BSA (Sigma-Aldrich, USA) in PBS with 0.1% Tween for 2 h in room temperature. The membranes were incubated overnight at 4 ℃ with the following primary antibodies: rabbit anti-mouse/rat primary antibodies at a concentration of 1:1000 for NDRG1, NDRG2, NDRG3, and NDRG4 (all from Cell Signaling Technology, USA); a rabbit anti-mouse/rat primary antibody at a concentration of 1:1000 for Lamin (Abcam, UK); and a rabbit anti-mouse/rat primary antibody at a concentration of 1:4000 for β-actin (Abcam, UK). The membranes were then incubated with peroxidase-conjugated goat anti-rabbit secondary antibodies (Boster, China) at a concentration of 1:100,000 for 1 h in room temperature. The blotted bands were visualized using an enhanced chemiluminescence Kit (Amersham Biosciences, USA) and a gel imaging system (5500; Tanon, China).

### Statistical analysis

All the results are represented as the mean ± standard deviation (SD). The data were analyzed using two-tailed Student’s *t* tests for two group comparisons or One-way analysis of variance (ANOVA) followed by the Newman-Keuls post-hoc tests for multiple group comparisons in the GraphPad Prism 5.01 software. Values of *P* < 0.05 were considered to be statistically significant.

## Electronic supplementary material

Appendix

## References

[1] Fletcher EL (2010). Mechanisms of photoreceptor death during retinal degeneration. Optom. Vis. Sci..

[2] Randolph SA (2014). Age-related macular degeneration. Workplace Health Saf..

[3] Hartong DT, Berson EL, Dryja TP (2006). Retinitis pigmentosa. Lancet.

[4] Organisciak DT, Vaughan DK (2010). Retinal light damage: mechanisms and protection. Prog. Retin. Eye Res..

[5] Chen YY, Liu SL, Hu DP, Xing YQ, Shen YN (2014). -methyl- N -nitrosourea-induced retinal degeneration in mice. Exp. Eye Res..

[6] Zhang B, Osborne NN (2006). Oxidative-induced retinal degeneration is attenuated by epigallocatechin gallate. Brain Res..

[7] Niwa M (2016). Retinal cell degeneration in animal models. Int. J. Mol. Sci..

[8] Tsubura A (2011). Review: animal models of N-Methyl-N-nitrosourea-induced mammary cancer and retinal degeneration with special emphasis on therapeutic trials. Vivo.

[9] Melotte V (2010). The N-myc downstream regulated gene (NDRG) family: diverse functions, multiple applications. FASEB J..

[10] Kovacevic Z, Richardson DR (2006). The metastasis suppressor, Ndrg-1: a new ally in the fight against cancer. Carcinogenesis.

[11] Hu W (2016). Emerging role of N-myc downstream-regulated gene 2 (NDRG2) in cancer. Oncotarget.

[12] Weiler M (2014). mTOR target NDRG1 confers MGMT-dependent resistance to alkylating chemotherapy. Proc. Natl Acad. Sci. USA.

[13] Anderson KJ, Russell AP, Foletta VC (2015). NDRG2 promotes myoblast proliferation and caspase 3/7 activities during differentiation, and attenuates hydrogen peroxide-But not palmitate-induced toxicity. FEBS Open Bio..

[14] Cui C, Lin H, Shi Y, Pan R (2017). Hypoxic postconditioning attenuates apoptosis via inactivation of adenosine A2a receptor through NDRG3-Raf-ERK pathway. Biochem. Biophys. Res. Commun..

[15] Kalaydjieva L (2000). N-myc downstream-regulated gene 1 is mutated in hereditary motor and sensory neuropathy-Lom. Am. J. Hum. Genet..

[16] Mitchelmore C (2004). NDRG2: a novel Alzheimer’s disease associated protein. Neurobiol. Dis..

[17] Yamamoto H (2011). NDRG4 protein-deficient mice exhibit spatial learning deficits and vulnerabilities to cerebral ischemia. J. Biol. Chem..

[18] Bassett EA, Wallace VA (2012). Cell fate determination in the vertebrate retina. Trends Neurosci..

[19] Takita S, Wada Y, Kawamura S (2016). Effects of NDRG1 family proteins on photoreceptor outer segment morphology in zebrafish. Sci. Rep..

[20] Flugge G, Araya-Callis C, Garea-Rodriguez E, Stadelmann-Nessler C, Fuchs E (2014). NDRG2 as a marker protein for brain astrocytes. Cell Tissue Res..

[21] Hirakawa M (2008). Age-related maculopathy and sunlight exposure evaluated by objective measurement. Br. J. Ophthalmol..

[22] Egger A (2012). PGC-1alpha determines light damage susceptibility of the murine retina. PLoS One.

[23] Tan E (2004). Expression of cone-photoreceptor-specific antigens in a cell line derived from retinal tumors in transgenic mice. Invest. Ophthalmol. Vis. Sci..

[24] O’Driscoll C, Doonan F, Sanvicens N, Messeguer A, Cotter TG (2011). A novel free radical scavenger rescues retinal cells in vivo. Exp. Eye Res..

[25] Sui BD (2017). Recipient glycemic micro-environments govern therapeutic effects of mesenchymal stem cell infusion on osteopenia. Theranostics.

[26] Sui B (2016). Mesenchymal progenitors in osteopenias of diverse pathologies: differential characteristics in the common shift from osteoblastogenesis to adipogenesis. Sci. Rep..

[27] Al-Ubaidi MR (2014). RGC-5: are they really 661W? The saga continues. Exp. Eye Res..

[28] Kanan Y, Moiseyev G, Agarwal N, Ma JX, Al-Ubaidi MR (2007). Light induces programmed cell death by activating multiple independent proteases in a cone photoreceptor cell line. Invest. Ophthalmol. Vis. Sci..

[29] Baker SA, Kerov V (2013). Photoreceptor inner and outer segments. Curr. Top. Membr..

[30] Tsuruma K (2012). Role of oxidative stress in retinal photoreceptor cell death in N-methyl-N-nitrosourea-treated mice. J. Pharmacol. Sci..

[31] Chen WJ (2017). Nrf2 protects photoreceptor cells from photo-oxidative stress induced by blue light. Exp. Eye Res..

[32] Song Y, Huang L, Yu J (2016). Effects of blueberry anthocyanins on retinal oxidative stress and inflammation in diabetes through Nrf2/HO-1 signaling. J. Neuroimmunol..

[33] Han MH (2017). Cytoprotective effects of esculetin against oxidative stress are associated with the upregulation of Nrf2-mediated NQO1 expression via the activation of the ERK pathway. Int. J. Mol. Med..

[34] Wang L (2008). NDRG2 is a new HIF-1 target gene necessary for hypoxia-induced apoptosis in A549 cells. Cell. Physiol. Biochem..

[35] Uehara N, Miki K, Tsukamoto R, Matsuoka Y, Tsubura A (2006). Nicotinamide blocks N-methyl-N-nitrosourea-induced photoreceptor cell apoptosis in rats through poly (ADP-ribose) polymerase activity and Jun N-terminal kinase/activator protein-1 pathway inhibition. Exp. Eye Res..

[36] Kiuchi K, Yoshizawa K, Shikata N, Matsumura M, Tsubura A (2002). Nicotinamide prevents N-methyl-N-nitrosourea-induced photoreceptor cell apoptosis in Sprague-Dawley rats and C57BL mice. Exp. Eye Res..

[37] Kiuchi K (2003). Functional rescue of N-methyl-N-nitrosourea-induced retinopathy by nicotinamide in Sprague-Dawley rats. Curr. Eye Res..

[38] Yu TY, Acosta ML, Ready S, Cheong YL, Kalloniatis M (2007). Light exposure causes functional changes in the retina: increased photoreceptor cation channel permeability, photoreceptor apoptosis, and altered retinal metabolic function. J. Neurochem..

[39] Fain GL, Lisman JELight (1999). Ca2+, and photoreceptor death: new evidence for the equivalent-light hypothesis from arrestin knockout mice. Invest. Ophthalmol. Vis. Sci..

[40] Chen Y, Perusek L, Maeda A (2016). Autophagy in light-induced retinal damage. Exp. Eye Res..

[41] Hao W (2002). Evidence for two apoptotic pathways in light-induced retinal degeneration. Nat. Genet..

[42] Zhang C (2005). Activation of microglia and chemokines in light-induced retinal degeneration. Mol. Vis..

[43] Puthussery T, Fletcher E (2009). Extracellular ATP induces retinal photoreceptor apoptosis through activation of purinoceptors in rodents. J. Comp. Neurol..

[44] Russo R (2016). Retinal ganglion cell death in glaucoma: exploring the role of neuroinflammation. Eur. J. Pharmacol..

[45] Himori N (2013). Critical role of Nrf2 in oxidative stress-induced retinal ganglion cell death. J. Neurochem..

[46] Park HY, Kim JH, Park CK (2012). Activation of autophagy induces retinal ganglion cell death in a chronic hypertensive glaucoma model. Cell Death Dis..

[47] Okuda T, Kokame K, Miyata T (2008). Differential expression patterns of NDRG family proteins in the central nervous system. J. Histochem. Cytochem..

[48] Shimauchi-Matsukawa Y, Aman Y, Tachibanaki S, Kawamura S (2008). Identification of differentially expressed genes in carp rods and cones. Mol. Vis..

[49] Yao L, Zhang J, Liu X (2008). NDRG2: a Myc-repressed gene involved in cancer and cell stress. Acta Biochim. Biophys. Sin..

[50] Vervoorts J, Luscher-Firzlaff J, Luscher B (2006). The ins and outs of MYC regulation by posttranslational mechanisms. J. Biol. Chem..

[51] Takahashi K (2012). Dexamethasone indirectly induces Ndrg2 expression in rat astrocytes. J. Neurosci. Res..

[52] Yamamura A (2013). Suppressed expression of NDRG2 correlates with poor prognosis in pancreatic cancer. Biochem. Biophys. Res. Commun..

[53] Chen N (2016). microRNA-21 contributes to orthodontic tooth movement. J. Dent. Res..

[54] Hu CH (2017). miR-21 deficiency inhibits osteoclast function and prevents bone loss in mice. Sci. Rep..

[55] Zhao S (2016). H2O2 treatment or serum deprivation induces autophagy and apoptosis in naked mole-rat skin fibroblasts by inhibiting the PI3K/Akt signaling pathway. Oncotarget.

[56] Donovan M, Cotter TG (2002). Caspase-independent photoreceptor apoptosis in vivo and differential expression of apoptotic protease activating factor-1 and caspase-3 during retinal development. Cell Death Differ..

[57] Frasson M (1999). Retinitis pigmentosa: rod photoreceptor rescue by a calcium-channel blocker in the rd mouse. Nat. Med..

[58] Ramchani-Ben Othman K, Cercy C, Amri M, Doly M, Ranchon-Cole I (2015). Dietary supplement enriched in antioxidants and omega-3 protects from progressive light-induced retinal degeneration. PLoS One.

[59] Liu C, Li Y, Peng M, Laties AM, Wen R (1999). Activation of caspase-3 in the retina of transgenic rats with the rhodopsin mutation s334ter during photoreceptor degeneration. J. Neurosci..

[60] Bode C, Wolfrum U (2003). Caspase-3 inhibitor reduces apototic photoreceptor cell death during inherited retinal degeneration in tubby mice. Mol. Vis..

[61] Chaum E (2003). Retinal neuroprotection by growth factors: a mechanistic perspective. J. Cell. Biochem..

[62] Joseph RM, Li T (1996). Overexpression of Bcl-2 or Bcl-XL transgenes and photoreceptor degeneration. Invest. Ophthalmol. Vis. Sci..

[63] Hahn P (2004). Deficiency of Bax and Bak protects photoreceptors from light damage in vivo. Cell Death Differ..

[64] Cao J (2016). Human umbilical tissue-derived cells rescue retinal pigment epithelium dysfunction in retinal degeneration. Stem Cells.

[65] Shirai H (2016). Transplantation of human embryonic stem cell-derived retinal tissue in two primate models of retinal degeneration. Proc. Natl Acad. Sci. USA.

[66] Wenzel A, Grimm C, Samardzija M, Reme CE (2005). Molecular mechanisms of light-induced photoreceptor apoptosis and neuroprotection for retinal degeneration. Prog. Retin. Eye Res..

[67] Shintani K, Shechtman DL, Gurwood AS (2009). Review and update: current treatment trends for patients with retinitis pigmentosa. Optometry.

[68] Keller C, Grimm C, Wenzel A, Hafezi F, Reme C (2001). Protective effect of halothane anesthesia on retinal light damage: inhibition of metabolic rhodopsin regeneration. Invest. Ophthalmol. Vis. Sci..

[69] Sieving PA (2001). Inhibition of the visual cycle in vivo by 13-cis retinoic acid protects from light damage and provides a mechanism for night blindness in isotretinoin therapy. Proc. Natl Acad. Sci. USA.

[70] Wang C, Liu B, Lu J, Zhang G, Lu A (2014). Strategies for combination of aptamer and targeted drug delivery. J. Nanosci. Nanotechnol..

[71] Jiang F (2015). Progress and challenges in developing aptamer-functionalized targeted drug delivery systems. Int. J. Mol. Sci..

[72] Jing H (2017). Declining histone acetyltransferase GCN5 represses BMSC-mediated angiogenesis during osteoporosis. FASEB J..

[73] Zheng CX (2018). Adipose mesenchymal stem cells from osteoporotic donors preserve functionality and modulate systemic inflammatory microenvironment in osteoporotic cytotherapy. Sci. Rep..

[74] Zheng C, Sui B, Hu C, Jin Y (2015). [Vitamin C promotes in vitro proliferation of bone marrow mesenchymal stem cells derived from aging mice]. Nan. Fang. Yi. Ke. Da. Xue. Xue. Bao..

[75] Lv YJ (2018). Resveratrol counteracts bone loss via mitofilin-mediated osteogenic improvement of mesenchymal stem cells in senescence-accelerated mice. Theranostics.

[76] Zhao P (2017). Anti-aging pharmacology in cutaneous wound healing: effects of metformin, resveratrol, and rapamycin by local application. Aging Cell..

[77] Sui B (2016). Allogeneic mesenchymal stem cell therapy promotes osteoblastogenesis and prevents glucocorticoid-induced osteoporosis. Stem Cells Transl. Med..

[78] Wang YJ (2018). Resveratrol enhances the functionality and improves the regeneration of mesenchymal stem cell aggregates. Exp. Mol. Med..

[79] Zhao P (2017). Anti-aging pharmacology in cutaneous wound healing: effects of metformin, resveratrol, and rapamycin by local application. Aging Cell..

[80] Zhou HS (2018). Lipopolysaccharide impairs permeability of pulmonary microvascular endothelial cells via Connexin40. Microvasc. Res..

