# Supplementary material for: NDRG2 suppression as a molecular hallmark of photoreceptor-specific cell death in the mouse retina
Source: Cell Death Discov. 2018 Sep 12;4:99. doi: 10.1038/s41420-018-0101-2 (PMC6135825; doi:10.1038/s41420-018-0101-2)
Supplement: Supplementary file 1 — Appendix [file 41420_2018_101_MOESM1_ESM.pdf]

Supplementary Information

NDRG2 suppression as a molecular hallmark of photoreceptor-specific cell death in the mouse retina

Running Title: NDRG2 contributes to photoreceptor homeostasis

Cheng-Biao Hu [1, 2, †], Bing-Dong Sui [3, 4, †], Bao-Ying Wang [1, 2, †], Gao Li [4, 5], Cheng-Hu Hu [3, 4], Chen-Xi Zheng [3, 4], Fang-Ying Du [1, 2], Chun-Hui Zhu [1, 2], Hong-Bo Li [1, 2], Yan Feng [1, 2], Yan Jin [3, 4, *] and Xiao-Rui Yu [1, 2, *]

[1] Department of Biochemistry and Molecular Biology, School of Basic Medical Sciences, Xi'an Jiaotong University Health Science Center, Xi'an, Shaanxi 710061, China.

[2] Key Laboratory of Environment and Genes Related to Diseases (Xi'an Jiaotong University), Ministry of Education, China.

[3] State Key Laboratory of Military Stomatology, Center for Tissue Engineering, Fourth Military Medical University, Xi'an, Shaanxi 710032, China.

[4] Xi'an Institute of Tissue Engineering and Regenerative Medicine, Xi'an, Shaanxi 710032, China.

[5] Department of stomatology, The people's Hospital of Zhangqiu City, Zhangqiu, Shandong 250200,China.

[†] These authors contributed equally to this work.

[*] Corresponding author

Prof. Xiao-Rui Yu

Department of Biochemistry and Molecular Biology, School of Basic Medical Sciences, Xi'an Jiaotong

University Health Science Center, No. 76 West Yanta Road, Xi'an, Shaanxi 710061, China.

E-mail: xiaoruiy@mail.xjtu.edu.cn; Tel/fax: +86-29-82655491.

Prof. Yan Jin

State Key Laboratory of Military Stomatology, Center for Tissue Engineering, Fourth Military Medical University, No. 145 West Changle Road, Xi'an, Shaanxi 710032, China.

E-mail: yanjin@fmmu.edu.cn; Tel: +86-29-84776472; Fax: +86-29-83218039.

Journal: Cell Death and Disease.

Supplementary information: 4 Supplementary Figures and 1 Supplementary Table.

Supplementary Figures and Legends

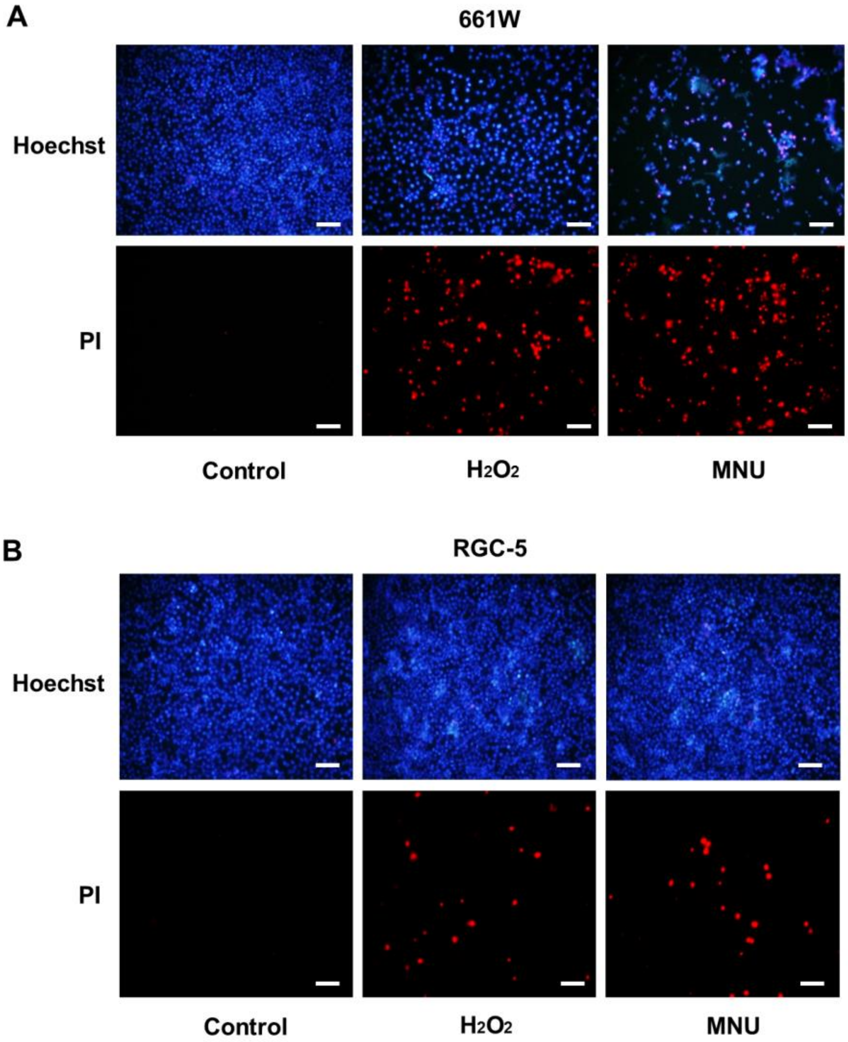

Supplementary Figure 1. Analysis of retinal cell death. Dead cells were positively stained by propidium iodide (PI) in the 661W (**A**) and RGC-5 (**B**) cell lines. Scale bar = 20 μm. *N* = 3 per group.

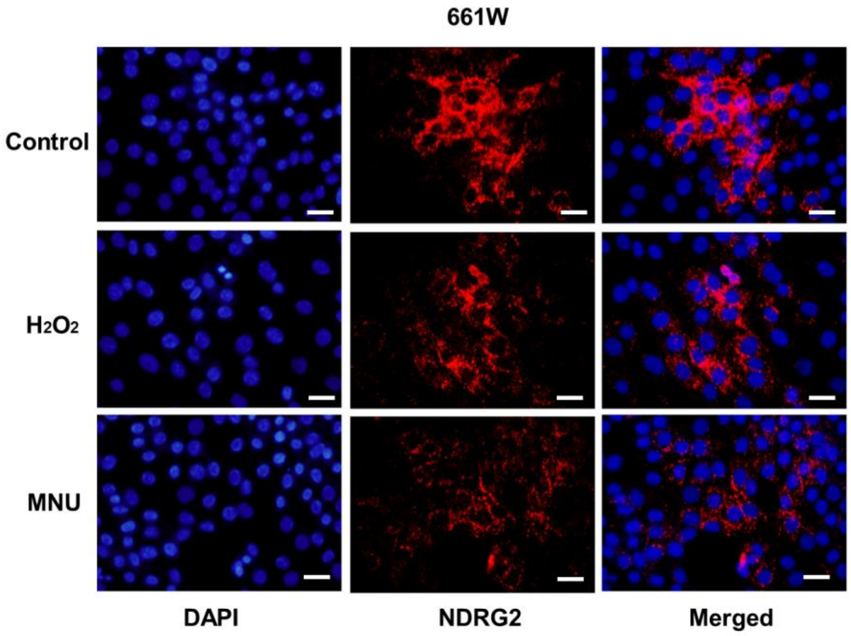

Supplementary Figure 2. Immunofluorescent staining of NDRG2 in the 661W photoreceptor cell line.

Scale bar = 2 μm. *N* = 3 per group.

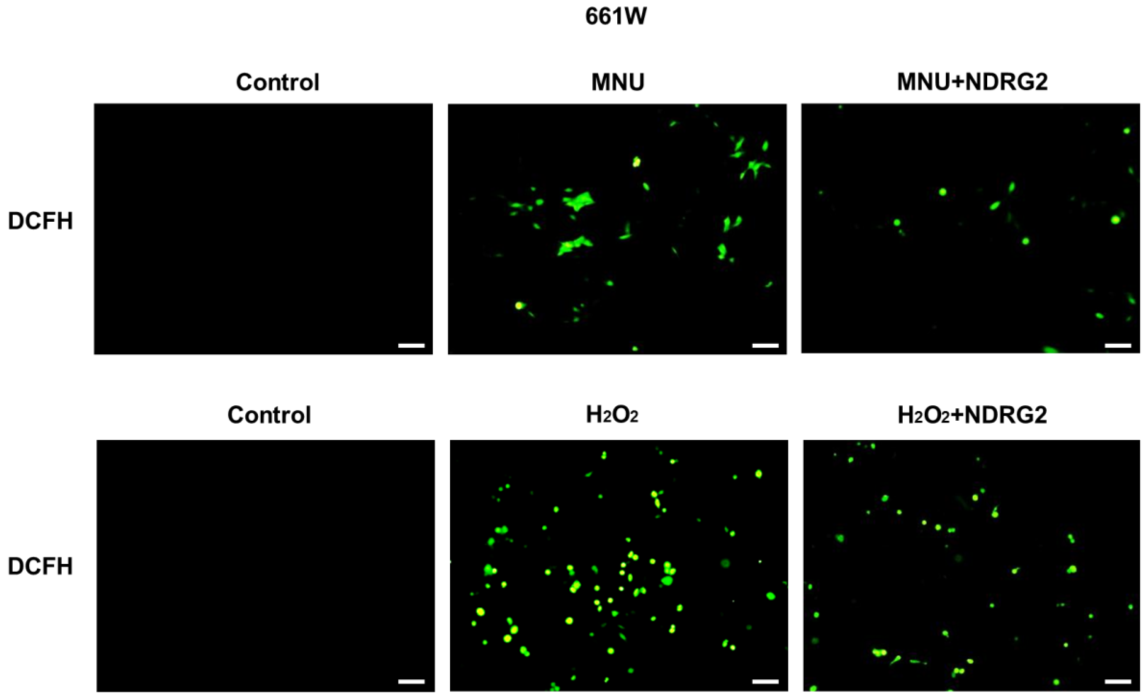

Supplementary Figure 3. Contents of reactive oxygen species (ROS) in the 661W photoreceptor cell line. 661W cells were overexpressed with NDRG2 in indicated groups. ROS was detected by the fluorescent dye Dichlorodihydrofluorescein Diacetate (DCFH). Scale bar = 1 μm. *N* = 3 per group.

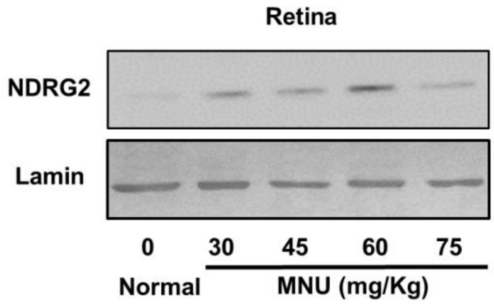

Supplementary Figure 4. Nuclear protein expression level of NDRG2 in the retinal tissue. Lamin was used as the internal control.

Supplementary Table 1. Primer sequences in the present study.

Gene	Primer sequences
β-actin	Forward: 5’-CTCTTTTCCAGCCTTCCTTCTT-3’
	Reverse: 5’-GAGGTCTTTACGGATGTCAACG-3’
NDRG1	Forward: 5’-CCTCAACGACATGAACCCGA-3’
	Reverse: 5’-TGCAAAGTGACAGTGTGGGT-3’
NDRG2	Forward: 5’-CGCGATATGCTCTGAACCAC-3’
	Reverse: 5’-TTCCTGGCTGAAAAGATGTCC-3’
NDRG3	Forward: 5’-CCGTATGTGCAGCTCAGTCA-3’
	Reverse: 5’-CTTGAGGTGGAGTGGGTTCG-3’
NDRG4	Forward: 5’-CAGCCATCCTCACCTACCAT-3’
	Reverse: 5’-GTGGCACACCACAAAGTGTT-3’
Nrf2	Forward: 5’-GGACATGGAGCAAGTTTGGC-3’
	Reverse: 5’-CAGCGGTAGTATCAGCCAGC-3’
Ho-1	Forward: 5’-TGACACCTGAGGTCAAGCAC-3’
	Reverse: 5’-ATCTTGCACCAGGCTAGCAG-3’
Nqo1	Forward: 5’-GGTAGCGGCTCCATGTACTC-3’
	Reverse: 5’-CCAGACGGTTTCCAGACGTT-3’
